# Detection of Methicillin Resistance in *Staphylococcus aureus* From Agar Cultures and Directly From Positive Blood Cultures Using MALDI-TOF Mass Spectrometry-Based Direct-on-Target Microdroplet Growth Assay

**DOI:** 10.3389/fmicb.2020.00232

**Published:** 2020-02-14

**Authors:** Ilka D. Nix, Evgeny A. Idelevich, Luise M. Storck, Katrin Sparbier, Oliver Drews, Markus Kostrzewa, Karsten Becker

**Affiliations:** ^1^Institute of Medical Microbiology, University Hospital Münster, Münster, Germany; ^2^Bruker Daltonik GmbH, Bremen, Germany; ^3^Friedrich Loeffler-Institute of Medical Microbiology, University Medicine Greifswald, Greifswald, Germany

**Keywords:** MALDI-TOF MS, direct-on-target microdroplet growth assay, antimicrobial susceptibility testing, rapid testing, blood culture, MRSA, *Staphylococcus aureus*

## Abstract

Matrix-assisted laser desorption/ionization time-of-flight-mass spectrometry (MALDI-TOF MS)-based direct-on-target microdroplet growth assay (DOT-MGA) was recently described as a novel method of phenotypic antimicrobial susceptibility testing (AST). Here, we developed the application of MALDI-TOF MS-based DOT-MGA for Gram-positive bacteria including AST from agar cultures and directly from positive blood cultures (BCs) using the detection of methicillin resistance as example. Consecutively collected, a total of 14 methicillin-resistant *Staphylococcus aureus* (MRSA) and 14 methicillin-susceptible *S. aureus* (MSSA) clinical isolates were included. Furthermore, a collection of MRSA challenge strains comprising different *SCCmec* types, *mec* genes, and *spa* types was tested. Blood samples were spiked with MRSA and MSSA and positive BC broth processed by three different methods: serial dilution of BC broth, lysis/centrifugation, and differential centrifugation. Processed BC broth was directly used for rapid AST using DOT-MGA. Droplets of 6 μl with and without cefoxitin at the EUCAST breakpoint concentration were spotted in triplicates onto the surface of a MALDI target. Targets were incubated in a humidity chamber, followed by medium removal and on-target protein extraction with formic acid before adding matrix with an internal standard as a quality control (QC). Spectra were acquired and evaluated using MALDI Biotyper software. First, tests were considered as valid, if the growth control achieved an identification score of ≥1.7. For valid tests, same score criterion was used for resistant isolates when incubated with cefoxitin. An identification score <1.7 after incubation with cefoxitin defined susceptible isolates. On-target protein extraction using formic acid considerably improved detection of methicillin resistance in *S. aureus* and DOT-MGA showed feasible results for AST from agar cultures after 4 h incubation time. Comparing the different processing methods of positive BC broth, lysis/centrifugation method with a final dilution step 10^–1^ of the 0.5 McFarland suspension resulted in best test performance after 4 h incubation time. Overall, 96.4% test validity, 100% sensitivity, and 100% specificity were achieved for detection of methicillin resistance in clinical isolates. All strains of the MRSA challenge collection were successfully tested as methicillin-resistant. This first study on Gram-positive organisms showed feasibility and accuracy of MALDI-TOF MS-based DOT-MGA for rapid AST of *S. aureus* from agar cultures and directly from positive BCs.

## Introduction

In times of raising antimicrobial resistance, rapid microbiological diagnostics is important to improve patients’ outcome (Trenholme et al., 1989; Barenfanger et al., 2008). If a timely effective antimicrobial therapy is missing, the mortality rate increases significantly (Kumar et al., 2006; Ferrer et al., 2014). Particularly in case of sepsis, a rapid and accurate detection of resistant pathogens is important (Banerjee et al., 2016; Dubourg et al., 2018; Idelevich et al., 2018a). Bloodstream infections are one of the most often cause of death (Singer et al., 2016). For routine sepsis diagnostics, several rapid molecular tests are available. However, these tests only detect the molecular resistance mechanism and do not give reliable information on the phenotypic resistance of the pathogen. Therefore, there is a great call for rapid phenotypic antimicrobial susceptibility testing (AST) in routine diagnostics (Doern, 2018; Dubourg et al., 2018; van Belkum et al., 2019). Implementation of matrix-assisted laser desorption/ionization time-of-flight mass spectrometry (MALDI-TOF MS) for identification (Idelevich et al., 2014b) and AST (Idelevich et al., 2014a) from positive blood culture (BC) broth shortly incubated on solid medium leads to an adjustment of empiric antibiotic therapy (Köck et al., 2017) and has already reached the routine microbiological diagnostics (Idelevich et al., 2019).

A MALDI-TOF MS-based direct-on-target microdroplet growth assay (DOT-MGA) was recently developed which allows a universal rapid AST (Idelevich et al., 2018b). It could also be shown that rapid phenotypic detection of resistance directly from positive BCs using DOT-MGA is feasible and accurate (Idelevich et al., 2018c). To enable the best possible therapy for the patient, it should be a goal to provide the results of susceptibility testing on the same day as it is now possible for pathogen identification (Idelevich and Becker, 2019). MALDI-TOF MS method provides a solid basis for a method that can do both: identification and rapid AST in one diagnostic workflow. Direct susceptibility testing from positive BCs with Gram-negative species by the MALDI-TOF MS-based DOT-MGA was previously established (Idelevich et al., 2018c). In this study, we investigate different processing methods of positive BC broth to establish the detection of methicillin resistance in *Staphylococcus aureus* directly from positive BCs using MALDI-TOF MS-based DOT-MGA, in addition to the rapid detection of methicillin resistance in *S. aureus* from cultures grown on solid medium. Methicillin-susceptible *S. aureus* (MSSA) and methicillin-resistant *S. aureus* (MRSA) isolates were chosen here as an example for Gram-positive bacteria because of the high importance of this pathogen in general and due to the special therapeutic and infection prevention challenges of its methicillin- and multi-resistance phenotypes (Köck et al., 2014; World Health Organization [WHO], 2017).

## Materials and Methods

### Bacterial Strains

For the study, 14 MRSA isolates were consecutively collected in the diagnostic laboratory of the Institute of Medical Microbiology, University Hospital Münster (Münster, Germany). Additionally, 14 consecutive MSSA isolates were collected and included into the study. To avoid copy isolates, only one isolate per patient was included. For genotypic characterization, proof of presence of *mecA* and *mecC* genes was done by GenoType MRSA (Hain Lifescience GmbH, Nehren, Germany) according to the manufacturer’s instructions. MSSA and MRSA isolates were genotyped based on sequencing of the protein A gene’s (*spa*) hypervariable region and *spa* types were assigned on the Ridom SpaServer^1^ curated by the SeqNet.org initiative (Mellmann et al., 2006). An ethical review process was not required for this study, as patient data were neither collected nor used in this *in vitro* experimental study.

Additionally, a challenge strain collection of highly diverse MRSA strains including various *SCCmec* types and *mec* genes and diverse *spa* types was selected applying isolates from the studies of Kriegeskorte et al. (2012), Schaumburg et al. (2012), and Becker et al. (2018). Overall, 16 isolates were chosen, comprising each two of *mecA* gene-positive isolates with *SCCmec* types I, II, III, IV, IVa, and V, respectively (Schaumburg et al., 2012), three *mecC* gene-positive isolates with *SCCmec* type XI (Kriegeskorte et al., 2012), and one plasmid-encoded *mecB*-positive isolate (Becker et al., 2018) (see Supplementary Table 2).

### Determination of Minimum Inhibitory Concentration

The minimum inhibitory concentration (MIC) of cefoxitin was determined for all isolates by broth microdilution reference method according to ISO standard 20776-1 (ISO, 2006) corresponding to the European Committee on AST (EUCAST, 2019a) and the guidelines of the Clinical and Laboratory Standards Institute (CLSI, 2018). Using cation-adjusted Mueller–Hinton broth (CAMHB, BD Diagnostic, Heidelberg, Germany), a final inoculum size of approximately 5 × 10^5^ cfu/ml was adjusted and confirmed by vital cell counting of serial dilutions onto tryptic soy agar (TSA) plates in triplicates after overnight incubation. Cefoxitin (TCI Deutschland, Eschborn, Germany) concentrations in a range of 0.25–128 mg/L were tested in double dilution steps. After 18 ± 2 h incubation at 35 ± 1°C the results were read. All tests were performed in triplicates and the median MIC was determined. MIC results were interpreted according to the EUCAST breakpoints (EUCAST, 2019a). MIC_50_, MIC_90_, and MIC ranges of consecutively collected clinical isolates were calculated for analysis. *S. aureus* ATCC 29213 reference strain was used as quality control (QC).

### MALDI-TOF MS-Based Rapid Antimicrobial Susceptibility Testing From Cultures Grown on Solid Medium

MALDI-TOF MS-based DOT-MGA was performed according to Idelevich et al. (2018b). In brief, bacterial suspension with standard turbidity of 0.5 McFarland (approx. 10^8^ cfu/ml) of an overnight culture on solid medium was prepared. The 0.5 McFarland suspension diluted 1:100 in CAMHB. A 96-well microtiter plate was prepared; 50 μl of the diluted bacterial suspension was added to 50 μl CAMHB as growth control. Additionally, 50 μl diluted bacterial suspension was added to 50 μl CAMHB containing cefoxitin, which results in a final inoculum size of approximately 5 × 10^5^ cfu/ml, which is the recommended inoculum for broth microdilution by ISO/EUCAST (ISO, 2006; EUCAST, 2019a). Final inoculum size was confirmed by vital cell counting of serial dilutions onto TSA plates in triplicate and counting of colonies after overnight incubation. The final concentration of cefoxitin was 4 mg/L, which is the breakpoint concentration dividing between susceptible and resistant isolates, according to EUCAST (2019a). For each isolate a growth control and a set-up containing cefoxitin were done in triplicates, with tests performed simultaneously on the same target. Six microliters was transferred from each well of the microtiter plate directly onto disposable MALDI target (MBT Biotarget 96, Bruker Daltonik GmbH, Bremen, Germany). For each time point a separate target was prepared. The targets were incubated in a plastic transport box (Bruker Daltonik GmbH, Bremen, Germany) where 4 mL of water was added to generate a humid atmosphere to avoid evaporation of microdroplets. The boxes were incubated for 3, 4, and 5 h at 35 ± 1°C. After incubation, the liquid medium on top of the sedimented bacteria was removed to avoid interference with broth ingredients during MALDI-TOF MS measurement. While carefully touching the droplets in a row simultaneously sidewise at its bottom on the target surface using small filter papers (Whatman^®^ Drying Block 556, Whatman GmbH, Dassel, Germany), the medium was removed due to capillary effects, as described in previous studies (Idelevich et al., 2018b), but using small filter papers instead of tissue wipes. The fully dried spots on the target were overlaid with 1 μl 70% formic acid to disrupt the cell membrane in order to achieve higher sensitivity and specificity for susceptibility detection. According to the standard protocol, the dried spots were overlaid with 1 μl MBT FAST Matrix (Bruker Daltonik GmbH, Bremen, Germany) solved in the standard solvent (Solution OS, LCH CHIMIE, Les Aires, France) according to the manufacturer’s instructions.

### MALDI-TOF MS-Based Rapid Antimicrobial Susceptibility Testing Directly From Positive Blood Cultures

#### Inoculation of Blood Culture Bottles

To simulate a bacteremia, 10 mL human blood (from KB, principal investigator, own blood donation for experimental *in vitro* purpose does not require this experiment to be reviewed or approved by the local ethics committee of the University of Münster) were spiked with a bacterial suspension to generate a bacteremia with approximately 10 cfu/ml. In brief, bacterial suspension with standard turbidity of 0.5 McFarland of an overnight culture on solid medium was prepared. The standardized bacterial suspension was diluted up to 10^–5^ in 1:10 dilution steps in CAMHB. 100 μl of dilution 10^–5^ were mixed with 10 ml blood. Disinfection of septum with 70% ethanol was done before and after injection. The spiked blood was injected into an aerobic plastic culture vial (BACTEC^TM^ Plus Aerobic/F, BD Diagnostic, Heidelberg, Germany) and BC bottles were incubated in a BACTEC^TM^ 9240 Automated BC System (BD Diagnostic, Heidelberg, Germany). Bacterial growth was detected using the fluorescent sensor technology and bottles were flagged positive when growth was detected. Time to positivity was automatically documented. The final inoculum size of approximately 10 cfu/ml was confirmed by vital cell counting of serial dilutions onto TSA plates in triplicate and counting of colonies after overnight incubation.

#### Processing of Positive Blood Cultures

**FIGURE 1:**
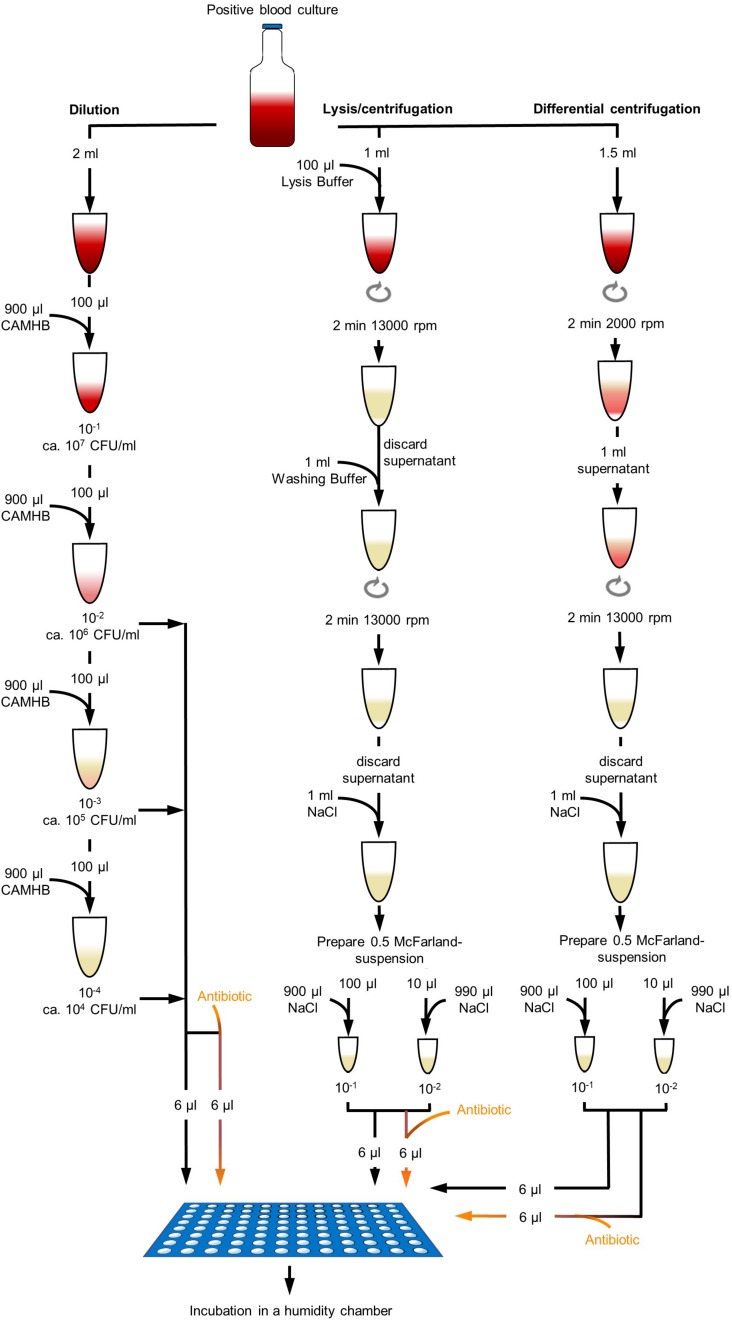
Different processing methods of positive blood culture broth. Dilution method was done with dilution steps up to 10^– 4^ in CAMHB. Dilutions were directly used for DOT-MGA. Lysis/centrifugation method was done according to the protocol of MBT Sepsityper kit (Bruker Daltonik GmbH, Bremen, Germany; version prior to introduction of rapid workflow and improved formulation), but only 100 μl lysis buffer was used. Differential centrifugation was performed with a first centrifugation step of 2 min at 2000 rpm and a second centrifugation step of 2 min at 13,000 rpm. Obtained pellets were resuspended and a standardized 0.5 McFarland suspension was prepared. Two different dilutions were used for DOT-MGA.

Blood culture bottles were removed from the incubator when flagged positive within the personnel’s working hours and processed immediately after removal. Preliminary experiments, done with six MRSA and six MSSA consecutively collected clinical isolates, used modified processing methods for isolation of bacteria from positive BCs for rapid AST, but could not reach satisfactory results. For preliminary experiments, each positive BC sample was processed by filtration/dilution, dilution, lysis/centrifugation, and differential centrifugation and DOT-MGA was incubated for 4, 5, and 6 h. Exact procedure of processing methods of preliminary experiments is described in Supplementary Data 1. To improve the results and processing methods of the preliminary experiments (Supplementary Table 1) and to analyze the best suitable method for isolation of bacteria from positive BCs for rapid AST in this present study, positive BC of 28 *S. aureus* consecutively collected clinical isolates and additionally 16 isolates of MRSA challenge strain collection described above were finally processed with three different methods: dilution, lysis/centrifugation, and differential centrifugation (Figure 1).

##### Dilution

Positive BC broth was diluted in decimal dilution steps up to 10^–4^ in CAMHB. Dilution steps 10^–2^,10^–3^, and 10^–4^ were used for DOT-MGA. Real bacterial concentration in positive BC broth was determined by vital cell counting of serial dilutions onto TSA plates in triplicate and counting of colonies after overnight incubation.

##### Lysis/centrifugation

For the lysis/centrifugation method, the MBT Sepsityper kit (Bruker Daltonik GmbH, Bremen, Germany) was used according to the manufacturer’s instructions (version prior to introduction of rapid workflow and improved formulation), but instead of 200 μl lysis buffer only 100 μl lysis buffer was used and the obtained pellet was suspended in 1 ml 0.9% NaCl. A bacterial suspension with standard turbidity of 0.5 McFarland was prepared. The standardized bacterial suspension was diluted 1:10 and 1:100 in CAMHB and both dilutions were used for DOT-MGA. The real bacterial concentration in test was determined by vital cell counting as described above.

##### Differential centrifugation

Differential centrifugation was used as a third method. To separate the blood cells from the bacterial cells, a first low speed centrifugation step was performed. 1.5 ml positive BC broth was centrifuged at 2000 rpm for 2 min. 1 ml of supernatant was transferred into a new reaction tube and centrifuged at 13,000 rpm for 2 min. Supernatant was discarded and the obtained pellet was suspended in 1 ml 0.9% NaCl. A bacterial suspension with standard turbidity of 0.5 McFarland was prepared. The standardized bacterial suspension was diluted 1:10 and 1:100 in CAMHB and dilutions were used for DOT-MGA. Real bacterial concentration in test was determined by vital cell counting as described above.

#### MALDI-TOF MS Direct-on-Target Microdroplet Growth Assay

Three different methods for processing of positive BC broth were performed and the resulting inoculum was used for rapid AST by DOT-MGA. Procedure of DOT-MGA was described above (see the section “MALDI-TOF MS-Based Rapid Antimicrobial Susceptibility Testing From Cultures Grown on Solid Medium”). In brief, 50 μl of cefoxitin solution in CAMHB was added to 50 μl of bacterial suspension in CAMHB in a well of a 96-well microtiter plate. The final concentration of cefoxitin was 4 mg/L. Additionally, 50 μl of bacterial suspension in CAMHB was added to 50 μl CAMHB as a growth control. For each sample, the growth control and the set-up containing cefoxitin were prepared in triplicate. Six microliters of droplets of each well of the microtiter plate was transferred directly on a hydrophilic spot of a disposable MBT Biotarget 96 (Bruker Daltonik GmbH, Bremen, Germany), resulting in three spots being inoculated with the sample with cefoxitin and three spots containing the growth control without cefoxitin for the corresponding sample. A separate target was prepared for each time point. The targets were incubated in a plastic transport box (Bruker Daltonik GmbH, Bremen, Germany) where 4 ml water was added to generate a humidity chamber. The boxes were incubated for 3, 4, 5, and 6 h at 35 ± 1°C. The microtiter plate was incubated for 18 ± 2 h at 35 ± 1°C too, as an additional control of each experiment. After incubation, the liquid medium was removed as described above and dried spots were treated with 1 μl 70% formic acid and overlaid with 1 μl MBT FAST Matrix (Bruker Daltonik GmbH, Bremen, Germany) solved in the standard solvent. MBT FAST Matrix contains an internal standard as a QC for spectra acquisition.

### Spectrum Acquisition and Categorization

MALDI-TOF MS measurement was performed using the MALDI Biotyper smart instrument (Bruker Daltonik GmbH, Bremen, Germany) and the flexControl software (Version 3.4, Bruker Daltonik GmbH, Bremen, Germany). Spectra were acquired with optimized instrument settings for DOT-MGA assay. Random walk and adapted acceptance criteria of the intensity were selected. The acquired spectra were analyzed using the MALDI Biotyper Compass Explorer (Version 4.1; Bruker Daltonik GmbH, Bremen, Germany) and matched against the BDAL database [Update Version 4.1 with 7854 Main Spectra (MSP) entries]. Spectra were categorized and interpreted as previously described according to the identification (score of ≥1.7)/no-identification (score of <1.7) evaluation (Idelevich et al., 2018b, c). Briefly, if the growth control without cefoxitin achieved an identification score of ≥1.7 for the tested isolate, the test was considered as valid. The test was invalid, if the score of the growth control was <1.7. Valid samples with cefoxitin and a score ≥1.7 resulted in a successful species identification and were interpreted as resistant. Valid samples with cefoxitin and a score <1.7 resulted in a failed identification and were interpreted as susceptible. In analogy to current approach for MIC determination, median score results of three spots were calculated and used for analysis.

### Data Evaluation

Detection of growth, i.e., score ≥ 1.7, as median result of three spots for the growth control was the criteria for a valid test, and test validity rate was calculated. Sensitivity of resistance detection was defined as the proportion of isolates correctly tested as resistant by DOT-MGA method among resistant isolates according to standard method. Specificity was defined as the proportion of isolates correctly tested among susceptible isolates determined by standard method. The relationship between two variables was expressed by the Pearson correlation coefficient (*r*) (Pearson, 1931, 1932). To test if calculated correlation is statistically significant (*p*), Student’s *t*-test (Student, 1908) was done and a significant level of α = 0.05 was determined.

### Spectrophotometric Measurement of Bacterial Growth by Optical Density

Bacterial growth of positive BC broth treated with different processing methods was analyzed by spectrophotometric measurement using a Multi-Mode Reader (Synergy HTX, BioTek, Bad Friedrichshall, Germany). Blood was spiked as described above and positive BC broth was processed (i) with differential centrifugation and a first centrifugation step of 2 min at 2000 rpm followed by a second centrifugation step of 2 min at 13,000 rpm as pictured above. (ii) The differential centrifugation method was modified with a first centrifugation step of 5 min at 2000 rpm. (iii) The MBT Sepsityper kit (Bruker Daltonik GmbH, Bremen, Germany; version prior to introduction of rapid workflow and improved formulation) was used according to the manufacturer’s instructions and (iv) the MBT Sepsityper kit protocol was modified and instead of 200 μl lysis buffer only 100 μl lysis buffer was used. The standardized bacterial inoculum (McFarland 0.5) was diluted 1:200, while 10 μl was added to 1990 μl CAMHB. Dilution was directly transferred to a microtiter plate for analysis of bacterial growth. Microtiter plate was incubated at 37°C for 18 h in the Multi-Mode Reader, measurement of optical density was performed every 10 min and before measurement the plate was shaken. Three wells of a microtiter plate were read in parallel and mean of optical density at 578 nm was calculated.

## Results

### Characterization of Bacterial Isolates With Standard Methods

Using reference AST method, the cefoxitin MIC_50_, MIC_90_, and MIC range of MSSA consecutively collected clinical isolates were 4, 4, and 2 to 4 mg/L, respectively. For consecutive clinical MRSA isolates, cefoxitin MIC_50_, MIC_90_, and MIC range were 128, >128, and 16 to >128 mg/L, respectively. The MIC of QC strain *S. aureus* ATCC 29213 was within the recommended range of 1–4 mg/L (EUCAST, 2019b). Phenotypic susceptibility results were confirmed by genotypic characterization. The *mecA* gene was detected in all consecutive clinical MRSA isolates while the *mecC* gene was not detected. Overall, 21 different *spa* types were detected. One isolate each within the 14 MSSA consecutively collected clinical isolates was *spa* type t002, t003, t012, t015, t021, t032, t084, t209, t315, t1043, t2078, or t2333. Two MSSA consecutively collected clinical isolates were *spa* type t091. One isolate each within the 14 consecutive clinical MRSA isolates was *spa* type t034 (livestock-associated MRSA), t045, t304, t437, t693, t4929, or t5857. Two MRSA each were *spa* type t003 or t011 (livestock-associated MRSA), respectively, and three MRSA isolates were *spa* type t032. MICs and *spa* type distribution for challenge strain collection isolates are demonstrated in Supplementary Table 2.

### MALDI-TOF MS Based DOT-MGA From Cultures Grown on Solid Medium

The accuracy of rapid detection of methicillin resistance in consecutively collected clinical isolates of *S. aureus* using MALDI-TOF MS-based DOT-MGA from solid medium overnight cultures are shown in Table 1. The best performance of MALDI-TOF MS-based DOT-MGA was observed after 5 h of incubation showing each 100% for sensitivity, specificity, and test validity. The 4-h incubation provided equal percentages of sensitivity and specificity for detection of methicillin resistance, but only 85.7% test validity. Final inoculum size of approximately 5 × 10^5^ cfu/ml was reached for all tested isolates (data not shown). MRSA challenge strain collection isolates were detected successfully after 5 or 6 h (Supplementary Table 3).

**TABLE 1:** Rapid AST from cultures grown on solid medium.

Incubation time	Validity^a^(%)	Sensitivity^b^(%)	Specificity^b^(%)
3 h	28.6	92.9	100
4 h	85.7	100	100
5 h	100	100	100

*Accuracy of detection of methicillin resistance in *S. aureus* (consecutively collected clinical isolates, *n* = 28) using MALDI-TOF MS-based DOT-MGA. ^*a*^Valid test – the growth control was detected (identification score ≥1.7 for the tested isolate). ^*b*^Calculated for valid tests.*

### MALDI-TOF MS-Based DOT-MGA Directly From Positive Blood Cultures

The mean determined bacterial concentration in inoculated blood samples was 9 CFU/ml (range, 2–15 CFU/ml). The time to positivity of BC bottles inoculated with consecutively collected clinical MSSA isolates was 673 min on average. Bottles inoculated with consecutive clinical MRSA isolates were flagged as positive on average after 702 min. BC bottles were removed from the incubator when flagged positive during the daily working hours and processed immediately after removal. The average times between positivity signal and processing for MSSA was 233 and for MRSA was 217 min, respectively. The mean bacterial concentration in positive BC was 1.5 × 10^9^ CFU/ml (range, 3.2 × 10^6^–8.7 × 10^9^ CFU/ml). It could be shown that shorter times to positivity were linked to higher bacterial concentrations in inoculated blood, see Supplementary Figure 1. Additionally, higher bacterial concentrations of positive BC broth were associated with shorter times to positivity. A relation between bacterial concentration of inoculated blood and bacterial concentration in positive BC broth could not be observed. Likewise, there was no connection between determined bacterial concentration in positive BC broth and time until processing after positivity signal, i.e., additional incubation after positivity (Supplementary Figure 1).

Using different processing methods for positive BC broth, the determined final bacterial concentration in test was different. Final real bacterial concentrations in test after processing positive BC broth with different methods are shown in Table 2. The mean inoculum size of lysis/centrifugation method (10^–1^ dilution) and differential centrifugation method (10^–1^ dilution) reached on average the recommended inoculum size of 5 × 10^5^ CFU/ml (ISO, 2006; CLSI, 2018). Serial dilution of positive BC broth (10^–3^) provided a mean inoculum size close to this recommended inoculum size as well. Performance of MALDI-TOF MS-based DOT-MGA for direct detection of methicillin resistance in *S. aureus* from positive BCs using different processing methods is shown in Table 3. After 4 h of incubation time, lysis/centrifugation method (10^–1^ dilution) showed best result: 100% accuracy of detection of methicillin resistance was achieved and test validity was 96.4%. After 6 h of incubation time, serial dilution of positive BC broth (10^–4^) and lysis/centrifugation method (10^–1^ dilution) showed similar accurate results at a test validity of 96.4%. Comparing these results to results of preliminary experiments for MALDI-TOF MS-based DOT-MGA directly from positive BCs, that were shown in Supplementary Table 1, a considerably improvement was shown. Using the non-modified lysis/centrifugation method in these preliminary experiments, no valid tests could be observed. Results of the non-modified differential centrifugation method in preliminary experiments showed lower validity (41.7%) compared to validity of the modified differential centrifugation method (96.4%) after 6 h incubation time. However, even the dilution method showed distinct improvement. All MRSA challenge strain collection isolates were successfully detected after processing and performing MALDI-TOF MS-based DOT-MGA directly from positive BC (Supplementary Table 4).

**TABLE 2:** Determined final bacterial concentrations in tests achieved with different processing methods of positive BCs (consecutively collected clinical isolates, *n* = 28).

		Final concentration in test (CFU/ml)		
		
Processing method	Dilution	Mean	Minimum	Maximum
Dilution	10^–2^	7.2 × 10^6^	1.6 × 10^4^	4.4 × 10^7^
	10^–3^	7.6 × 10^5^	1.6 × 10^3^	4.4 × 10^6^
	10^–4^	7.6 × 10^4^	1.6 × 10^2^	4.4 × 10^5^
Lysis/centrifugation	10^–2^	3.8 × 10^4^	4.8 × 10^3^	3.6 × 10^5^
	10^–1^	3.8 × 10^5^	4.8 × 10^4^	3.6 × 10^6^
Differential centrifugation	10^–2^	3.0 × 10^4^	2.3 × 10^1^	1.2 × 10^5^
	10^–1^	3.0 × 10^5^	2.3 × 10^2^	1.2 × 10^6^

**TABLE 3:** Performance of MALDI-TOF MS DOT-MGA for direct detection of methicillin resistance in *Staphylococcus aureus* from positive BCs (consecutively collected clinical isolates, *n* = 28).

		3 h			4 h			5 h			6 h		
					
Processing method	Dilution	Validity^a^(%)	Sensitivity^b^(%)	Specificity^b^(%)	Validity^a^(%)	Sensitivity^b^(%)	Specificity^b^(%)	Validity^a^(%)	Sensitivity^b^(%)	Specificity^b^(%)	Validity^a^(%)	Sensitivity^b^(%)	Specificity^b^(%)
Dilution	10^–2^	96.4	100	78.6	100	100	85.7	100	100	92.9	100	100	92.9
	10^–3^	78.6	85.7	92.9	82.1	92.9	92.9	100	92.9	92.9	100	100	92.9
	10^–4^	32.1	92.9	100	78.6	85.7	100	85.7	85.7	100	**96.4**	**100**	**100**
Lysis/	10^–1^	75.0	85.7	92.9	**96.4**	**100**	**100**	**89.3**	**100**	**100**	**96.4**	**100**	**100**
centrifugation	10^–2^	7.1	85.7	–	46.4	100	100	71.4	100	100	89.3	100	100
Differential centrifugation	10^–1^	60.7	92.9	100	82.1	92.9	100	85.7	100	92.9	96.4	100	92.9
	10^–2^	3.6	100	–	50.0	100	100	53.6	100	100	67.9	92.9	100

*^*a*^Valid test – the growth control was detected (identification score ≥1.7 for the tested isolate). ^*b*^Calculated for valid tests. ^*c*^The values in bold indicate results with best test performance.*

## Discussion

For septic patients, a rapid AST directly from positive BCs would reduce time to beginning of an effective antimicrobial therapy and enhance patients’ outcome in comparison to standard susceptibility testing (Kumar et al., 2006). MALDI-TOF MS’ principal feasibility as a method for rapid AST could be shown in studies before (Lange et al., 2014). The recently developed DOT-MGA presents a universal phenotypic rapid AST based on MALDI-TOF MS (Idelevich et al., 2018b). MALDI-TOF MS-based DOT-MGA is able to perform identification and AST within few hours and could easily be implemented in routine laboratory workflows. To accelerate in particular the diagnostics of sepsis, direct susceptibility testing from positive BCs with Gram-negative species by the MALDI-TOF MS-based DOT-MGA was recently established (Idelevich et al., 2018c). Idelevich et al. (2018c) could show that rapid detection of carbapenem non-susceptibility in *Enterobacterales* directly from positive BCs was possible within 4 h using the MALDI-TOF MS-based DOT-MGA.

In this study, we focused on the rapid detection of methicillin resistance in *S. aureus* using MALDI-TOF MS, that is the first study of using MALDI-TOF MS-based DOT-MGA for Gram-positive species, such as *S. aureus*. Previous studies investigated the use of MALDI-TOF MS for this purpose by comparing acquired spectra of MSSA strains to spectra of MRSA strains to identify unique mass peaks. They found different mass peaks that were applied to differentiate MSSA and MRSA obtaining different sensitivities and specificities of this procedure (Edwards-Jones et al., 2000; Bernardo et al., 2002; Du et al., 2002; Wang et al., 2013). Josten et al. (2014) identified a small peptide (PSM-*mec*) produced by *agr*-positive strains that is part of the genomes of health care-associated MRSA. This peptide can be detected in the MALDI-TOF MS spectra (2415 Da) and could be used for rapid identification of this subgroup of MRSA with high specificity and sensitivity. However, a universal approach to determine methicillin susceptibility is still missing. The newly introduced MALDI-TOF MS-based DOT-MGA is a universal phenotypic assay that offers the opportunity to distinguish between susceptible and non-susceptible isolates in the presence of an antibiotic agent. Here, to discriminate between MSSA and MRSA, isolates were incubated in the presence of cefoxitin and growth or no growth was analyzed.

To accelerate the detection of MRSA strains, we focused on the rapid detection of methicillin resistance in *S. aureus* directly from positive BCs in this study. To process positive BC broth, three different methods were used: dilution, lysis/centrifugation, and differential centrifugation. Preliminary experiments to this study applied the same processing approach that Idelevich et al. (2018c) used for detection of carbapenem non-susceptibility in *Enterobacterales.* Here, results showed (Supplementary Table 1) that dilution method was the best to detect methicillin resistance in *S. aureus* directly from positive BC broth using MALDI-TOF MS-based DOT-MGA. Lysis/centrifugation and differential centrifugation method revealed less validity for detection of methicillin resistance in *S. aureus*. Due to this, analysis of bacterial growth by spectrophotometric measurement of optical density for isolates of positive BC broth treated with different processing methods was done and results showed that there was a delayed beginning of bacterial growth (Supplementary Figure 2). When treating positive BC broth with lysis/centrifugation method using MBT Sepsityper (Bruker Daltonik GmbH, Bremen, Germany) standard protocol (version prior to introduction of rapid workflow and improved formulation) and adding 200 μl lysis buffer to 1 ml BC broth according to the manufacturer’s instructions instead of adding only 100 μl lysis buffer to 1 ml BC broth, there was a delayed beginning of bacterial growth of up to 4 h. Idelevich et al. (2018c) demonstrated the performance of the lysis/centrifugation method for processing positive BC broth, when using the MBT Sepsityper standard protocol adding 200 μl lysis buffer to 1 ml blood for direct testing of carbapenem non-susceptibility in *Enterobacterales* using MALDI-TOF MS-based DOT-MGA. Gram-positive bacteria seem to be more sensitive against lysis buffer of MBT Sepsityper comparing Gram-negative bacteria. Due to the different cell wall structure *S. aureus* showed higher susceptibility compared to *Enterobacterales* isolates.

Additionally, we compared two different differential centrifugation methods (Supplementary Figure 2) one with a first centrifugation step of 2 min at 2000 rpm and the other with a first centrifugation step of 5 min at 2000 rpm to sediment the blood cells. After transferring the supernatant into a new centrifugation tube, bacteria were sedimented for 2 min at 13,000 rpm for both methods. Results showed that there was a delayed beginning of bacterial growth of 2 h when using a first centrifugation step of 5 min at 2000 rpm instead of 2 min. A probable explanation could be that Gram-positive cocci like to cluster and clustered cells become heavier, heavier cells were more sedimented when using a first centrifugation step of 5 min instead of 2 min at 2000 rpm. Lin et al. (2017) performed differential centrifugation method of positive BCs to identify species using MALDI-TOF MS and could show that identification of Gram-negative bacteria showed better results compared to Gram-positive species after differential centrifugation of positive BC broth.

Direct identification of species from positive BC broth using MALDI-TOF MS was introduced several years ago and different processing methods to treat the positive BC broth were tested (La Scola and Raoult, 2009; Christner et al., 2010; Moussaoui et al., 2010; Stevenson et al., 2010; Ferreira et al., 2011; Klein et al., 2012; Zhou et al., 2017). For choosing the best processing method of positive BC broth in our present study it is important that in contrast to classical MALDI-TOF MS identification, for DOT-MGA vital cells are needed for susceptibility testing. Regarding the results of previous experiments dilution method, lysis/centrifugation method with less lysis buffer compared to the manufacturer’s protocol and differential centrifugation method were chosen to have less impact on the bacterial growth for susceptibility testing and were chosen for this study. Considering, that MALDI-TOF MS identification results of Gram-positive species achieved better results when performing an on-target lysis with formic acid before adding matrix (Haigh et al., 2011), this formic acid on-target lysis step was also performed in this present study.

The study has shown that the rapid phenotypic AST using MALDI-TOF MS-based DOT-MGA for detection of methicillin resistance in subcultivated *S. aureus* on solid medium is successful within 4 h with a validity of 85.7% (Table 1), even successful detection of resistance in all MRSA challenge strains could be shown (Supplementary Table 3). Further results for rapid detection of methicillin resistance in *S. aureus* directly from positive BCs using MALDI-TOF MS-based DOT-MGA could reduce time to results in comparison to the standard susceptibility testing method from subcultivated colonies on solid medium for effective and fast sepsis diagnostics. Results of this presented study showed best performance of MALDI-TOF MS-based DOT-MGA for detection of methicillin resistance in *S. aureus* using modified lysis/centrifugation protocol and using a 10^–1^ dilution of McFarland 0.5 suspension to inoculate the assay (Table 3). Additionally, all MRSA challenge strains were detected successfully after processing the positive BC broth (Supplementary Table 4). Regarding the determined bacterial concentrations, the mean final inoculum was within the recommended inoculum according to EUCAST (5 × 10^5^ CFU/ml) if the standardized inoculum was diluted 1:10 after processing the positive BC broth with lysis/centrifugation (3.8 × 10^5^ CFU/ml) or differential centrifugation method (3.0 × 10^5^ CFU/ml). After dilution of the positive BC broth, the recommended inoculum according to EUCAST was achieved with the 10^–3^ dilution (7.6 × 10^5^CFU/ml). Overall, best results of DOT-MGA in this presented study were achieved with the inoculum recommended by EUCAST (5 × 10^5^ CFU/ml), which is a valid guideline for susceptibility testing. To give a general recommendation for inoculum density for DOT-MGA further analyses are needed.

**FIGURE 2:**
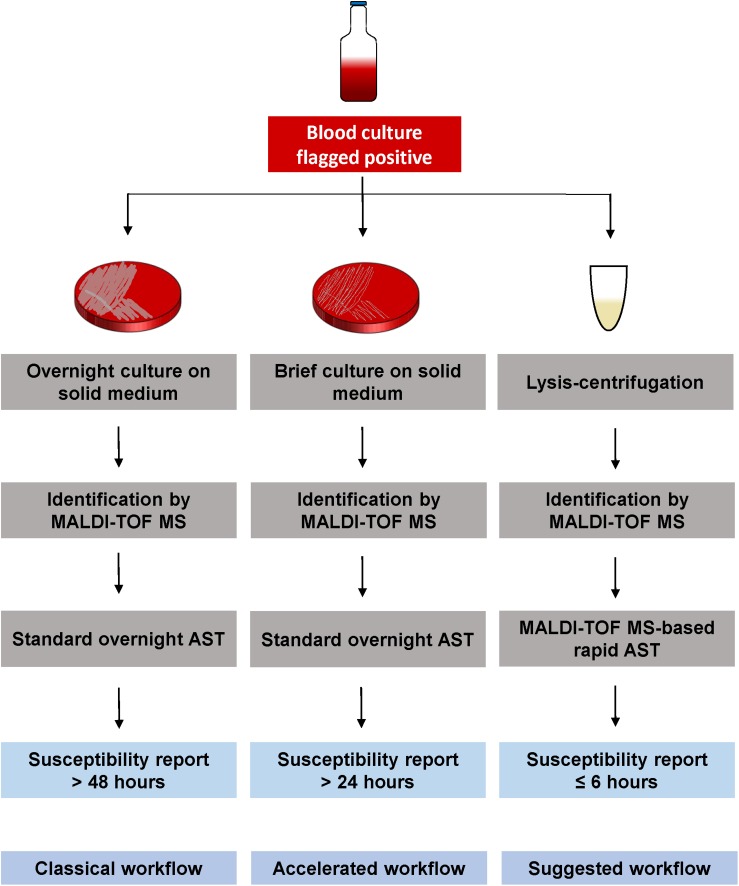
Different workflows for susceptibility testing of positive BCs. Classical workflow: Overnight sub-cultivation of positive BC broth, followed by identification of the pathogen using MALDI-TOF MS. An overnight susceptibility testing is performed and susceptibility results are finalized approx. 2 days after BC was flagged positive. Accelerated workflow: Shortly incubated solid medium cultures (2–6 h) are used for MALDI-TOF MS identification, followed by an overnight susceptibility testing from the same shortly incubated cultures. Susceptibility results are available the day after BC was flagged positive. Suggested workflow: Positive BC broth is processed by the lysis/centrifugation method, using the modified MBT Sepsityper protocol, followed by the MALDI-TOF MS identification. The obtained pellet (without protein extraction) is used for MALDI-TOF MS-based rapid AST. Susceptibility results are available the same day BC was flagged positive within <6 h.

To accelerate the diagnostic of sepsis, direct susceptibility testing from positive BCs needs to become a suitable method in routine laboratories. A simultaneous identification and susceptibility testing, after previous Gram classification, would reduce time, and an earlier start of an effective antimicrobial therapy could decrease mortality rate. MALDI-TOF MS-based DOT-MGA has the potential to further accelerate sepsis diagnostics, because it is characterized by minor hands-on-time and offers an easy and cost-efficient alternative way of rapid AST. A further advantage of this novel method is its ability to detect contaminations, because the correct identification of the growth control is a validity criterion along with the sufficient growth. An internal standard in the used MBT FAST matrix ensured a QC for spectra acquisition. As with other routine methods, identification and susceptibility testing by MALDI-TOF MS is hardly possible with mixed pathogens in the BC bottle. In this case, sub-culturing on solid medium is required to get a pure culture for identification and susceptibility testing. Since an increasing number of clinical microbiology laboratories apply MALDI-TOF MS instruments for rapid identification, rapid AST by MALDI-TOF MS could easily be integrated in daily diagnostic workflow. Our suggested workflow is shown in Figure 2 with lysis/centrifugation method using the modified MBT Sepsityper protocol for species identification by MALDI-TOF. After identification, the MALDI-TOF MS-based AST is performed with the obtained pellet. Final susceptibility results will be available in <6 h, starting with the positive flagged BC and including total hands-on-time of half an hour. According to the standard workflow in the majority of routine diagnostics laboratories, positive BC would be subcultivated on solid medium and, after an overnight incubation, identification is done by MALDI-TOF MS. This is followed by standard AST, which also requires overnight incubation. Some laboratories integrated the identification by MALDI-TOF MS from shortly incubated cultures on solid medium according to Idelevich et al. (2014b) to reduce time to result. Standard workflow would take up to 2 days until susceptibility results are available, whereas results will be available within 24 h using brief cultures on solid medium. Rapid AST by MALDI-TOF MS DOT-MGA would provide susceptibility results the same day BC was flagged positive. MALDI-TOF MS identification before initiation of AST provides precise guide to the choice of appropriate antibiotic panels to be tested.

## Conclusion

The first investigation of MALDI-TOF MS DOT-MGA for Gram-positive bacteria demonstrated the feasibility and accuracy of DOT-MGA from agar cultures and directly from positive BCs for detection of methicillin resistance in *S. aureus* after 4 h of incubation. To standardize and optimize test conditions and evaluation criteria, further research is needed. An optimized evaluation algorithm must be developed to guarantee the reliable performance of this new rapid AST assay. Furthermore, studies with larger amount of genetically diverse isolates from different locations elsewhere in the world should follow to develop a standardized assay for clinical diagnostics. Additionally, using this universal phenotypic assay for simultaneous rapid AST of several antibiotics in parallel would be in focus of further studies.

## Data Availability Statement

All datasets generated for this study are included in the article/Supplementary Material.

## Author Contributions

IN, EI, LS, and KB designed the experiments. LS performed the preliminary experiments. IN performed the experiments. IN, EI, KS, OD, and MK designed and analyzed specific MALDI-TOF MS instrument settings for experiments. IN, EI, KS, OD, and KB analyzed the data. IN wrote the manuscript with input from EI, LS, KS, OD, MK, and KB. All authors reviewed and edited the manuscript.

## Conflict of Interest

EI and KB are inventors of the patent application which is owned by the University of Münster and licensed to Bruker Daltonik GmbH, Bremen, Germany. KS, OD, and MK are employees of Bruker Daltonik GmbH, Bremen, Germany. The remaining authors declare that the research was conducted in the absence of any commercial or financial relationships that could be construed as a potential conflict of interest.

## Footnotes

1 http://spaserver.ridom.de
