# Supplementary material for: Detection of Methicillin Resistance in Staphylococcus aureus From Agar Cultures and Directly From Positive Blood Cultures Using MALDI-TOF Mass Spectrometry-Based Direct-on-Target Microdroplet Growth Assay
Source: Front Microbiol. 2020 Feb 14;11:232. doi: 10.3389/fmicb.2020.00232 (PMC7033577; doi:10.3389/fmicb.2020.00232)
Supplement: Supplementary file 1 [file Data_Sheet_1.PDF]

Supplementary Material

Supplementary Data 1.

Processing of positive blood cultures in preliminary experiments.

Preliminary experiments to the presented study used different processing methods for isolation of bacteria from positive BCs for rapid AST by MALDI-TOF MS-based DOT-MGA, but couldn't reach satisfactory results. Each positive BC sample was processed by filtration/dilution, dilution, lysis/centrifugation and differential centrifugation as described in the following. Six MRSA and six MSSA isolates were tested.

Filtration/dilution.
Positive BC broth was filtered through a sterile 5 µm syringe filter (Sartorius, Göttingen, Germany) and following by decimal dilution steps up to 10^{-4} in CAMHB. Dilution steps 10^{-2}, 10^{-3} and 10^{-4} were used for DOT-MGA. Real bacterial concentration was determined by vital cell counting of serial dilutions onto TSA plates in triplicate and counting of colonies after overnight incubation.

Dilution.
Positive BC broth was diluted in decimal dilution steps up to 10^{-4} in CAMHB. Dilution steps 10^{-2}, 10^{-3} and 10^{-4} were used for DOT-MGA. Real bacterial concentration was determined as described above.

Lysis/centrifugation.
For the lysis/centrifugation method the MBT Sepsityper (Bruker Daltonik; version prior to introduction of rapid workflow and improved formulation) was used according to the manufacturer's instructions. A bacterial suspension with standard turbidity of 0.5 McFarland was prepared. The standardized bacterial suspension was diluted 1:100 in CAMHB was used for DOT-MGA. The real bacterial concentration in test was determined by vital cell counting as described above.

Differential centrifugation.
For differential centrifugation, a first low speed centrifugation step was performed. 1.5 ml positive BC broth was centrifuged at 2000 rpm for 5 min. The supernatant was then centrifuged at 13000 rpm for 2 min. Supernatant was discard and the obtain pellet was suspended in 1 ml 0.9% NaCl. A bacterial suspension with standard turbidity of 0.5 McFarland was prepared and diluted 1:100 in CAMHB, to use for DOT-MGA. Real bacterial concentration in test was determined by vital cell counting as described above, likewise.

Four different methods for processing of positive BC broth were applied and the resulting inoculum was used for rapid AST by the DOT-MGA. DOT-MGA was described above according to Idelevich et al. (2018b). Three different time points (4, 5 and 6 hours) were tested for those described processing methods and MALDI-TOF MS based DOT-MGA for direct detection of methicillin resistance in *S. aureus* from positive BCs (n = 12).

Supplementary Table 1. Results of preliminary experiments using different processing methods. Performance of MALDI-TOF MS DOT-MGA for direct detection of methicillin resistance in *Staphylococcus aureus* from positive BCs (consecutively collected clinical isolates, n=12).

**Processing method**		**4 hours**			**5 hours**			**6 hours**		
		**Validity**[a]	**Sensitivity**[b]	**Specificity**[b]	**Validity**[a]	**Sensitivity**[b]	**Specificity**[b]	**Validity**[a]	**Sensitivity**[b]	**Specificity**[b]
**Filtration/dilution**	**10^{-2}**	33.3%	100%	100%	33.3%	100%	100%	50.0%	83.3%	100%
	**10^{-3}**	16.7%	100%	-	25.0%	100%	100%	33.3%	100%	100%
	**10^{-4}**	0.0%	-	-	0.0%	-	-	16.7%	100%	100%
**Dilution**	**10^{-2}**	**91.7%**	**83.3%**	**100%**	**91.7%**	**100%**	**100%**	**100%**	**100%**	**100%**
	**10^{-3}**	83.3%	100%	100%	83.3%	100%	100%	91.7%	100%	100%
	**10^{-4}**	50.0%	100%	100%	50.0%	100%	100%	83.3%	100%	100%
**Lysis/centrifugation**		0.0%	-	-	0.0%	-	-	0.0%	-	-
**Differential centrifugation**		0.0%	-	-	8.3%	-	100%	41.7%	83.3%	100%

[a] Valid test – the growth control was detected (identification score ≥ 1.7 for the tested isolate).
[b] Calculated for valid tests.
[c] The values in bold indicate results with best test performance.

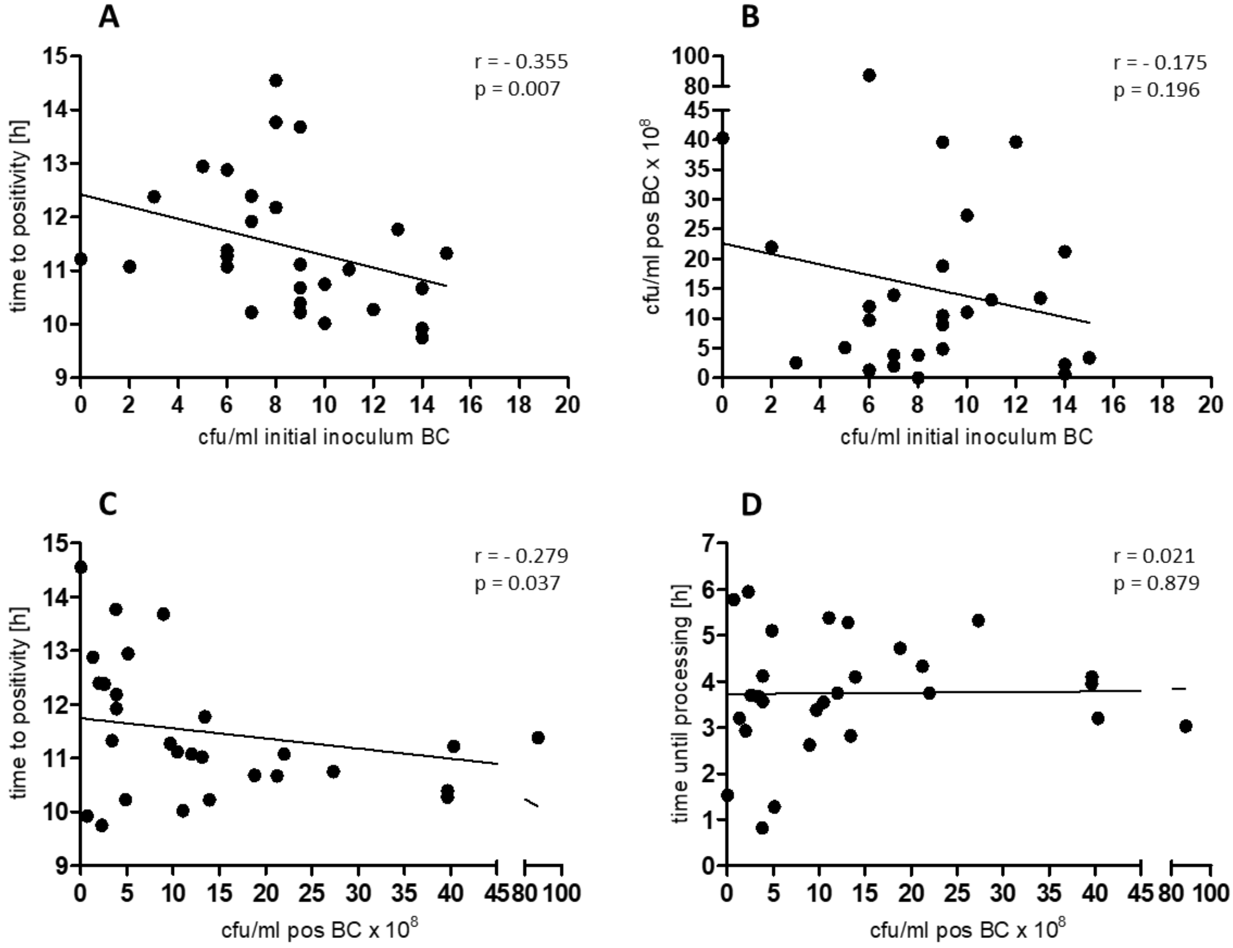

Supplementary Figure 1. Correlation of real bacterial concentration of initial inoculum or positive BC and time to positivity or time until processing. Calculation of correlation coefficient *r* and statistical significance *p*-value. *r* can vary from -1 to +1; values near -1 mean a strong negative relationship and values near +1 mean a strong positive relationship between two variables. Values near 0 mean no correlation between two variables. Calculated correlation is statistically significant (p), if $p \leq 0.05$. A significant correlation was shown between real bacterial concentration in inoculated blood and the time to positivity (A). Additionally, a significant correlation was demonstrated between real bacterial concentration in positive BC broth and time to positivity (C). No significant correlation could be shown between real bacterial concentration in positive BC broth and time until processing (D) and real bacterial concentration in inoculated blood and real bacterial concentration in positive BC broth (B).

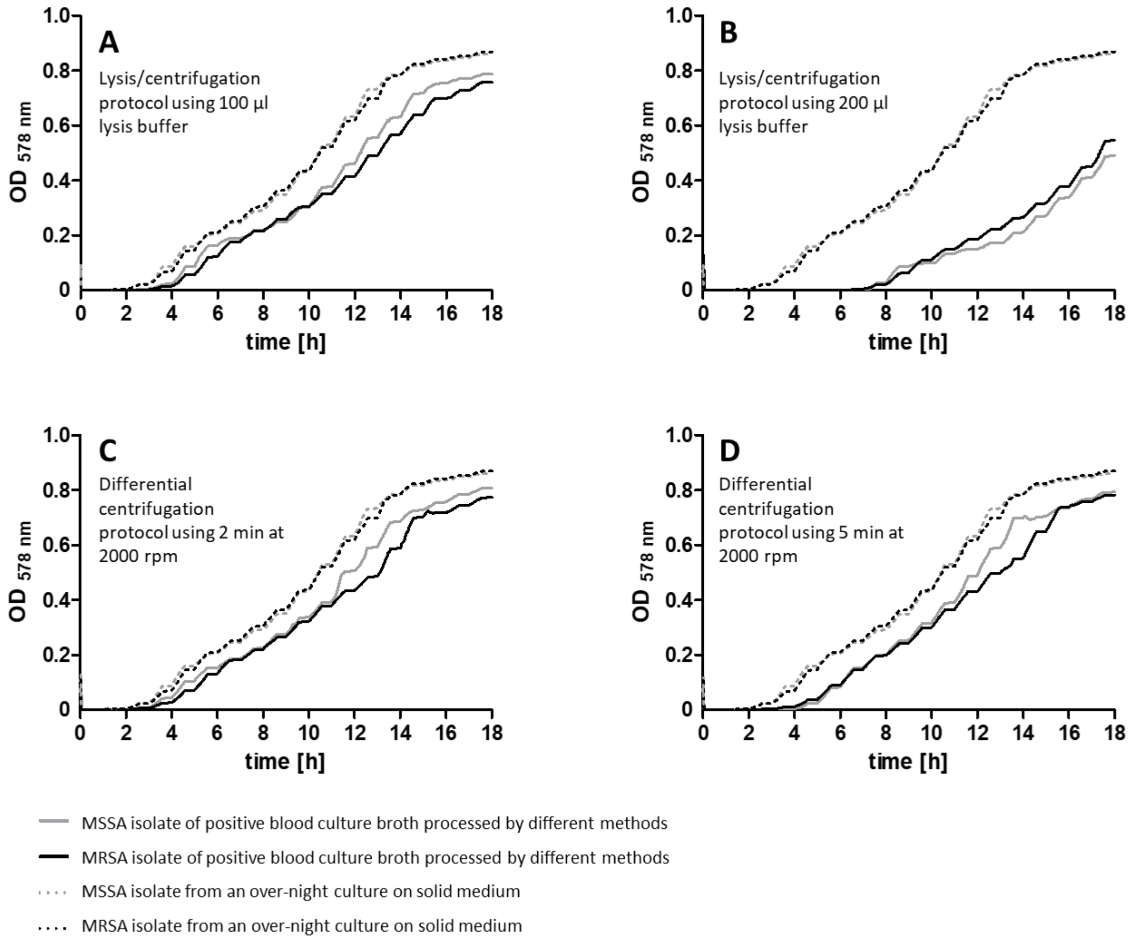

Supplementary Figure 2. Spectrophotometric measurement of bacterial growth by optical density for isolates of positive BC broth treated with different processing methods. Delayed beginning of growth could be shown for isolates of positive BC broth proceed with lysis/centrifugation method using MBT Sepsityper (Bruker Daltonik; version prior to introduction of rapid workflow and improved formulation) standard protocol according to the manufacturer's instructions (B) in contrast to the lysis/centrifugation method using only 100 µl lysis buffer (A). Threating positive BC broth with differential centrifugation method and a first centrifugation step of 5 min at 2000 rpm (D) showed a delayed beginning of growth in contrast to a first centrifugation step of 2 min at 2000 rpm (C), respectively.

Supplementary Table 2. Characterization of challenge strain collection isolates.

Strain[a]	*mec* gene	SCC*mec* type	*spa* type	Reference	Median MIC (mg/L)
**1-14**	*mecA*	I	t032	Schaumburg et al., 2012	32
**2-6**	*mecA*	I	t001	Schaumburg et al., 2012	64
**3-9**	*mecA*	II	t002	Schaumburg et al., 2012	64
**1-1**	*mecA*	II	t003	Schaumburg et al., 2012	32
**35-16**	*mecA*	III	t037	Schaumburg et al., 2012	32
**20-34**	*mecA*	III	t425	Schaumburg et al., 2012	64
**13-2**	*mecA*	IV	t038	Schaumburg et al., 2012	> 128
**15-34**	*mecA*	IV	t022	Schaumburg et al., 2012	64
**8-15**	*mecA*	IVa	t008	Schaumburg et al., 2012	64
**22-8**	*mecA*	IVa	t064	Schaumburg et al., 2012	> 128
**28-22**	*mecA*	V	t034	Schaumburg et al., 2012	> 128
**19-47**	*mecA*	V	t011	Schaumburg et al., 2012	32
**UKM4229**	*mecB*	None (plasmidal)	t091	Becker et al., 2018	32
**1-2010**	*mecC*	XI	t843	Kriegeskorte et al., 2012	16
**12-2007**	*mecC*	XI	t978	Kriegeskorte et al., 2012	32
**15-2010**	*mecC*	XI	t1535	Kriegeskorte et al., 2012	32

[a] With the exception of ovine strain 15-2010, all strains were isolated from humans.

Supplementary Table 3. Results of rapid AST from cultures grown on solid medium. Accuracy of detection of methicillin resistance in *S. aureus* (challenge strain collection, n = 16) using MALDI-TOF MS-based DOT-MGA.

Incubation time	Validity[a]	Correct detection[b]
**3 hours**	6.3%	0%
**4 hours**	87.5%	85.7%
**5 hours**	93.6%	93.3%
**6 hours[c]**	100%	100%

[a] Valid test – the growth control was detected (identification score ≥ 1.7 for the tested isolate).
[b] Correct detection of resistance (identification score ≥ 1.7 for the tested isolate) – calculated for valid tests.
[c] 6 hours incubation time was added for isolates that showed no growth after 5 hours.

Supplementary Table 4. Results of MALDI-TOF MS DOT-MGA for direct detection of methicillin resistance in *S. aureus* from positive BCs (challenge strain collection, n=16).

Processing method	Dilution	3 hours		4 hours		5 hours		6 hours	
		Validity[a]	Correct detection[b]	Validity[a]	Correct detection[b]	Validity[a]	Correct detection[b]	Validity[a]	Correct detection[b]
**Dilution**	**10^{-2}**	**100%**	**93.8%**	**100%**	**100%**	**100%**	**100%**	**100%**	**93.8%**
	**10^{-3}**	68.8%	72.7%	100%	93.8%	100%	100%	93.8%	100%
	**10^{-4}**	18.8%	0%	62.5%	70%	87.5%	85.7%	93.8%	93.3%
**Lysis/centrifugation**	**10^{-1}**	56.3%	55.6%	75.0%	83.3%	87.5%	92.9%	**100%**	**93.8%**
	**10^{-2}**	0%	-	31.3%	20.0%	68.8%	45.5%	81.3%	84.6%
**Differential centrifugation**	**10^{-1}**	**93.8%**	**93.3%**	**100%**	**100%**	**100%**	**100%**	**100%**	**100%**
	**10^{-2}**	0%	-	56.3%	55.6%	81.3%	76.9%	100%	93.8%

[a] Valid test – the growth control was detected (identification score ≥ 1.7 for the tested isolate).
[b] Correct detection of resistance (identification score ≥ 1.7 for the tested isolate) – calculated for valid tests.
[c] The values in bold indicate results with best test performance.
